# Headache from clinically confirmed hemicrania continua arising from the sternocleidomastoid muscle: a case report

**DOI:** 10.1186/s12883-021-02219-3

**Published:** 2021-05-03

**Authors:** Osnat Wende, Shira Markowitz

**Affiliations:** 1 Pain Unit, Department of Anesthesiology and Critical Care, Hadassah Medical Organization and Faculty of Medicine, Hebrew University of Jerusalem, Jerusalem, Israel; 2 Department of Neurology, Shaare Zedek Medical Center, Faculty of Medicine, Hebrew University of Jerusalem, Jerusalem, Israel

**Keywords:** Hemicrania continua, trigeminal autonomic cephalalgia, Trigeminal cervical complex, Cervical Myofascial pain, Case report

## Abstract

**Background:**

A patient with a history of cluster headaches, now in remission, presented with confirmed hemicrania continua that resolved with a local anaesthetic injection into the Sternocleidomastoid (SCM) muscle. To the best of our knowledge, this is the first reported case of a trigeminal autonomic cephalalgia arising from a soft tissue source in the neck.

**Case presentation:**

A 66-year-old man with a history of cluster headaches presented with a six-month history of a new constant right-sided headache. The new headaches were associated with tearing and redness of the right eye and responded to indomethacin, thus meeting the International Classification of Headache Disorders (ICHD-3) diagnostic criteria for hemicrania continua. The history and physical examination suggested a cervical source of the headache arising from the ipsilateral SCM muscle. Injection of the muscle with 1% lidocaine resulted in the elimination of the pain for 1 month without indomethacin.

**Conclusions:**

Due to the convergence of trigeminal, cervical and autonomic nerve fibres, various combinations of headache syndromes can result. This case report demonstrates how a meticulous examination is a crucial component of headache evaluation. Treatment directed to this muscle spared this patient further daily indomethacin and associated side effects.

## Background

The intricate and overlapping sensory and autonomic nerve supply to the head and face can result in headache syndromes presenting as unilateral headache with autonomic features. We present a case demonstrating that this type of headache can also be referred from a soft tissue structure in the neck. Our patient’s clinical presentation, which met the International Classification of Headache Disorders (ICHD-3) criteria for hemicrania continua [1, 2], is unique in its complete response to injection of local anaesthetic into the sternocleidomastoid (SCM) muscle. More common conditions, such as tension-type headache and headache with migraine-like features, can potentially be referred from cervical tissues [1, 3]. To the best of our knowledge, there have been no reports of any of the trigeminal autonomic cephalalgias arising from a cervicogenic soft tissue source. Rarely have trigeminal autonomic cephalalgias (TACs) been reported to derive from spinal cervicogenic sources of headache, as defined by the International Headache Society, though these were not from soft tissue sources [4, 5].

## Case presentation

A 66-year-old retired married, male patient was seen in the pain clinic in mid-2014 complaining of classic episodic cluster headaches for the previous 10 years. During this time, he had pain-free periods lasting for about 6 months. Headaches occurred up to 8 times a day, starting as a short stabbing pain in the medial aspect of his right eye and gradually spreading to the entire right side of his face, vertex and upper back of the head. The pain attacks would last between 45 and 75 min; he was completely pain-free between attacks. The pain felt ‘like his head was going to explode’ and was unbearable. The pain was associated with rhinorrhea in the right nostril and obvious conjunctival injection in the right eye without oedema. The patient was extremely restless and absolutely nothing he could do would relieve the pain. He was treated with a greater occipital nerve block comprising 40 mg methylprednisolone and 20 mg bupivacaine. The nerve block afforded him complete pain relief within minutes and lasted for 12 months. The block was repeated on two occasions, each time providing relief for a further 6 months. Subsequently, the frequency and intensity of the headaches subsided and by mid-2016 he was free of any headache for the next 2.5 years. Apart from a past history of an abdominal aortic aneurysm which was operated on, he was otherwise healthy. His family history was unremarkable.

The patient presented to the pain clinic again in July 2019 after suffering for 6 months from a new constant stabbing pain radiating from the back of the right side of his head to the right side of both the vertex and forehead, right ear and towards the jaw. Numbness and paresthesias were also present in this distribution. He had no neck pain. In addition, the intermittent tearing and conjunctival injection of the right eye that he previously had with his cluster headaches had become continuous and the eyelid was also edematous. He had excessive rhinorrhea. He often felt restless, particularly at night. The basal Numerical Rating Scale Pain Score (NRS) was 6 out of 10. Pain exacerbations that reached an NRS score of 9 out of 10, occurred 2–3 times a day and lasted 20–30 min. They were occasionally exacerbated by neck movements.

On physical examination, his right eye was mildly teary, with conjunctival injection and slight oedema of the eyelid. He had hyperesthesia over the right vertex and right side of his forehead. The neurological examination was otherwise normal. Passive extension of the neck was extremely limited and reproduced the severe exacerbations of his pain. Palpation of the right SCM muscle not only elicited tenderness but exacerbated the referred pain. This raised the suspicion that the SCM muscle was a source of the headache. Therefore, 30 mg of 1% lidocaine was injected into the right SCM muscle. The intention was to perform a diagnostic injection with a short-acting local anaesthetic to determine if the pain arose from the SCM. No other muscles were injected. Within 4 min of the injection, the severity of his headache decreased from a pain score of 7 out of 10 to 0. The numbness across his forehead and vertex disappeared. Neck range of motion became painless and much improved. The right-sided tearing and conjunctival injection began to subside, followed later by the resolving eyelid oedema. Relief of symptoms was complete for 4 weeks and then partially relapsed.

He returned to the pain clinic in September 2019 and, as he requested, received another injection. The right SCM muscle was just as tender as it was in July and palpation of it exacerbated the pain. This time the muscle was injected with 20 mg lidocaine and 10 mg bupivacaine. His pain completely resolved for a further 2 months.

In mid-December, when the headache relapsed, a second specialist in both neurology and pain relief medicine recommended that the patient start on a low dose of indomethacin, 25 mg three times a day, and then increase the dose slowly. Co-interventions, including further injections, were withheld in order to determine the efficacy of the indomethacin and so as not to undermine the diagnosis of hemicrania continua. The pain was relieved within 24 h of the indomethacin trial, confirming the diagnosis of hemicrania continua. There was no need to increase the dose, as his headache was eliminated. It was decided to discontinue the indomethacin due to the onset of severe gastric side effects. Six days later, by which time the headaches had completely relapsed, the patient was then given another injection of 20 mg lidocaine and 10 mg bupivacaine into the right SCM muscle. Once again, his pain was eliminated. During visits to the pain clinic for unrelated low back pain in June and October 2020, and February 2021, he reported that both his continuous headache as well as his cluster headaches were still in complete remission.

Our patient much preferred to have periodic injections than suffer from indomethacin-related side effects. The injections he received had no side effects and afforded him lasting relief.

## Discussion and conclusions

The ICHD-3 classification of headache disorders defines hemicrania continua as a persistent, strictly unilateral continuous headache, usually associated with prominent, ipsilateral cranial parasympathetic autonomic features, such as conjunctival injection and/or lacrimation, nasal congestion and/or rhinorrhea and eyelid oedema. Furthermore, the headache is absolutely responsive to indomethacin. These features were present in our patient. For the full ICHD-3 diagnostic criteria of hemicrania continua, the reader is referred to the ICHD-3 classification of headache disorders [1] or their website [2].

We propose that there may be converging sensory mechanisms in this patient whereby the headache is perpetuated or instigated from a soft tissue source in the neck, eliciting his hemicrania continua headache. Various types of headaches can result from the convergence of the C1–3 afferent spinal nerve fibres with trigeminal tract neurons forming the trigeminal cervical complex [6, 7]. This convergence helps explain why the soft tissues of the neck such as the SCM are capable of causing a pain referral pattern to the face.

The SCM muscle is innervated by spinal nerves C2–3. When this muscle is either injured, strained or harbours painful trigger points, a variety of clinical presentations, including headache, may develop. The SCM muscle has both a sternal and a clavicular (cleido) head. According to Travell and Simons, the sternal head refers pain only to the ipsilateral side of the head and face [8] as in this case. It must be stressed that the SCM muscle was repeatedly tender each time the pain relapsed.

Our findings a) that both active and passive neck movements exacerbated the headache, b) of painful limitation of neck movements and c) that palpation of the tender ipsilateral SCM muscle elicited exacerbation of the referred pain in the distribution of his headaches, all raised our suspicion that the SCM muscle was a potential source of the headache. The SCM, being a neck flexor, can trigger the headache when it is stretched by extension of the neck. We then confirmed our suspicions by injecting local anaesthetic into the SCM muscle which resulted in complete elimination of the pain for between 1 and 14 months without indomethacin.

This patient’s headache could perfectly fit well into 2 categories, as classified by the IHCD-3. All criteria for hemicrania continua were met, including the elimination of the pain with indomethacin. In addition, this headache fulfils the criteria of headache attributed to cervical myofascial pain syndrome, as summarized in Appendix A11.2.5 of the IHCD-3 [9]. Whenever the pain relapsed, trigger points in the right SCM were felt to be tender and palpation of them elicited referred pain.

The strengths of this case report are that this patient had all essential clinical features pertaining to two of the classes within the ICHD-3; a) hemicrania continua and b) referred pain from cervical myofascial pain syndrome. The main limitation was that the patient was not given indomethacin immediately upon his first visit for his hemicrania continua pain. An MRI of the brain and cervical spine for evaluation of the constant pain was not performed. However, because the pain was completely relieved for at least 1 month with an injection into soft tissue, the authors found no reason to pursue this.

This case report demonstrates the importance of taking a thorough history and performing a physical examination that includes the neck when evaluating patients with headaches, irrespective of the suspected diagnosis. This is particularly relevant when evaluating head and facial pain due to the intricate and converging sensory nerve supply to these regions. Prompt methodical diagnosis of headache may enable safe, tolerable and effective treatments to be applied in lieu of medications that are well known to be associated with severe side effects and risks.

## Data Availability

A copy of the medical notes is available and can be translated, de-identified, and submitted if requested.

## Abbreviations

ICHD-3: International Classification of Headache Disorders, 3rd Edition
NRS: Numerical Rating Scale
SCM: Sternocleidomastoid
TAC: Trigeminal autonomic cephalalgia
